# Glycohydrolases in the central nervous system: the role of GBA2 in the neuronal differentiation

**DOI:** 10.1186/2193-1801-4-S1-P42

**Published:** 2015-06-12

**Authors:** Maura Samarani, Nicoletta Loberto, Valentina Murdica, Domitilla Schiumarini, Simona Prioni, Alessandro Prinetti, Sandro Sonnino, Rosaria Bassi, Massimo Aureli

**Affiliations:** Department of Medical Biotechnology and Translational Medicine, Università degli Studi di Milano, Italy

**Keywords:** Sphingolipids, Neuronal differentiation, GBA2

Mammalian neurodevelopment is characterized by qualitative and quantitative changes in plasma membrane glycosphingolipids due to a fine regulation of their metabolic pathways. While the biosynthetic pathway is largely studied scant is the information available on the catabolic one. For this reason, I studied the activity of the main glycohydrolases expressed in the central nervous system during neuronal differentiation. To this purpose the activities of the principal glycohydrolases involved in glycosphingolipid catabolism have been evaluated in different experimental models such as brains and cerebella of mouse at different ages and neuronal cell cultures (immortalized mouse neuronal cell lines GN11 and GT1-7, primary cultures of mouse cerebellar granule cells and human neuroblastoma cells SH-SY5Y). The results obtained indicate that the process of neuronal differentiation is associated with a marked increase in the activities of all the glycohydrolases evaluated, in particular, the activity of the non-lysosomal β-glucosylceramidase GBA2 undergoes the most relevant increase representing the prevalent form of β-glucocerebrosidase in mature neurons. In order to evaluate the possible role of GBA2 in the neuronal differentiation, SH-SY5Y cells have been stably trasfected for GBA2 overexpression. Cells overexpressing GBA2 acquired a neuronal phenotype and showed a significant increase in ceramide levels. These results are in line with literature data that demonstrate the involvement of ceramide in the neuronal differentiation. Therefore, it is possible to hypothesize that the hydrolysis of glucosylceramide to ceramide, catalyzed by GBA2 at the plasma membrane level, has a functional role in the neuronal differentiation process. Collectively these findings suggest that GBA2 may represent a possible neuronal marker and demonstrate for the first time its direct involvement in the neuronal differentiation.

